# Effect of wheelchair design on wheeled mobility and propulsion efficiency in less-resourced settings

**DOI:** 10.4102/ajod.v6i0.342

**Published:** 2017-09-08

**Authors:** Christopher J. Stanfill, Jody L. Jensen

**Affiliations:** 1 International Society of Wheelchair Professionals, Washington, D.C., United States; 2 Department of Kinesiology and Health Education, University of Texas at Austin, United States

## Abstract

**Background:**

Wheelchair research includes both qualitative and quantitative approaches, primarily focuses on functionality and skill performance and is often limited to short testing periods. This is the first study to use the combination of a performance test (i.e. wheelchair propulsion test) and a multiple-day mobility assessment to evaluate wheelchair designs in rural areas of a developing country.

**Objectives:**

Test the feasibility of using wheel-mounted accelerometers to document bouts of wheeled mobility data in rural settings and use these data to compare how patients respond to different wheelchair designs.

**Methods:**

A quasi-experimental, pre- and post-test design was used to test the differences between locally manufactured wheelchairs (push rim and tricycle) and an imported intervention product (dual-lever propulsion wheelchair). A one-way repeated measures analysis of variance was used to interpret propulsion and wheeled mobility data.

**Results:**

There were no statistical differences in bouts of mobility between the locally manufactured and intervention product, which was explained by high amounts of variability within the data. With regard to the propulsion test, push rim users were significantly more efficient when using the intervention product compared with tricycle users.

**Conclusion:**

Use of wheel-mounted accelerometers as a means to test user mobility proved to be a feasible methodology in rural settings. Variability in wheeled mobility data could be decreased with longer acclimatisation periods. The data suggest that push rim users experience an easier transition to a dual-lever propulsion system.

## Introduction

### Problem statement

The number of different wheelchair designs being distributed around the world is growing at an accelerating pace, whereas information about the performance of these designs under different settings of user and terrain is lacking. Although typically prescribed by medical personnel, the wheelchair provided is often poorly fitted to the user and inappropriate for the setting in which the user lives. The result is the waste of millions of dollars, lost resources to local governments and institutions and harm to patients. It is important to understand how patients are responding to these wheelchair designs to fine-tune the assessment and product prescription phase.

Alternative propulsion systems (e.g. hand cycle, single lever, etc.) have been shown to improve biomechanical and physiological efficiency during short performance tests (Mukherjee & Samanta 2001b; Van der Woude, Dallmeijer & Janssen 2001; Winter et al. 2010, 2012). What is unknown is whether these alternative systems improve wheeled mobility over an extended period of time, outside of a testing condition. Changes in user movement behaviour can be detected in great detail using these validated methods for measuring wheeled mobility. Outcomes from these data can then yield information about the possibility of alternative designs promoting easier wheelchair travel in rural settings. New technology has made quantification of performance easier to achieve in rural settings. Wheel-mounted accelerometers allow us to quantify bouts of individual mobility, over an extended period of time, outside of a controlled testing condition. These accelerometers capture the rate at which the velocity of the chair changes. Attaching them to a wheelchair, then, allows us to determine how patients use their wheelchair and how their use may change when transitioning to an alternative wheelchair design.

The purpose of this study was to fill the void in the wheelchair performance literature by determining the effect of design (i.e. propulsion system) on bouts of mobility and propulsion efficiency over an extended period of time in less-resourced settings.

### Literature review

The premise of wheelchair design is to improve the mobility of the user (Van der Woude et al. 2001). Wheelchair use in less-resourced areas highlights the interaction between wheelchair design and mobility, as individuals in these communities face barriers because of inadequate infrastructure. The pursuit of improved mobility, however, is about more than just distance travelled. Mobility is also correlated to health outcomes with research showing that mobility is associated with decreased risks for developing secondary conditions such as diabetes, heart disease and obesity (Warms, Belza & Whiney 2007). Thus, researching wheeled mobility over an extended period of time, rather than through brief performance tests, maps the wheelchair user interface in a manner that provides insight into frequency, duration and overall usage. This information helps practitioners and designers understand how products are being utilised, and this information can then drive functional change.

Devices created specifically for use in rural areas of developing countries are designed to promote increased levels of wheeled mobility with consideration of environmental barriers (e.g. unpaved surfaces). The performance benefit of all-terrain wheelchairs is well documented from both a physiological (Cooper et al. 2008a; Mukherjee & Samanta 2001, 2001b; Winter et al. 2010) and biomechanical (Mukherjee & Samanta 2001, 2001b; Van der Woude, De Groot & Janssen 2006; Winter et al. 2010) perspective. Overall, tricycle (both single lever and bimanual hand cycle) models are more biomechanically and physiologically efficient when compared with push rim designs. However, push rim wheelchairs can still be found in rural areas because these designs are easier to manoeuvre when compared with their much larger tricycle counterparts.

Evaluation criteria in many of these studies include use of a peak performance course (Mukherjee & Samanta 2001a; Winter et al. 2010). Only a single study reports measurements on a course designed to simulate activities of daily living (Winter et al. 2012). Research taking place in semi-controlled performance test course settings cannot be considered generalisable to the overall effect of the wheelchair on the user. Routhier, Vincent and Desrosiers (2003) argue that performance courses (e.g. standardised obstacle courses) are better utilised in a rehabilitation setting where a patient must focus on goal-driven tasks, whereas product evaluation is best conducted during patients’ daily life activities (i.e. outside of a performance test setting).

The advantage of collecting information during daily life activities suggests that evaluation of wheelchair design effectiveness is best completed when testing in applied settings (i.e. natural environments). Use of wheeled mobility as a dependent measure accomplishes this goal by yielding comprehensive movement data that are collected via a wheel-mounted accelerometer. This method of research has yet to be used for the evaluation of wheelchair designs in less-resourced settings. Therefore, the logical progression of user performance research is to focus on the bigger picture, which is studying wheeled mobility during daily life activities. Wheel-mounted accelerometers can be used with an established performance test, such as the wheelchair propulsion test (WPT) (Askari et al. 2013), to provide more detail about the wheelchair user interface. The combination of wheeled mobility and propulsion efficiency can yield a detailed story about wheelchair-related activity and fill the void that currently exists within evaluation of the wheelchair user interface in the developing world.

#### Evaluating wheeled mobility

Dearwater et al. (1985) and Janssen et al. (1994) are credited for being pioneers in wheeled mobility measurement, but the primary limitation to their approach was the use of an odometer measurement for mobility. This approach can only measure distance travelled and does not provide information on *how* the individual is moving throughout a given period of time. Data logging systems (i.e. accelerometers) enable researchers to track displacement data in real time, which can then be converted into frequency, duration and distance. This approach serves as the foundation for contemporary wheeled mobility research. Tolerico et al. (2007) were the first to use mobility trackers, beyond that of odometers, on manual wheelchair users in an applied setting. Researchers mounted a series of reed switches and magnets on wheel spokes, which acted as sensors for collecting mobility data in an uncontrolled environment.

This placement provides valuable information on angular acceleration, but is also non-invasive to the user, which improves the quality of data being collected.

Cooper et al. (2008b) were the first to use wheel-mounted accelerometers for quantifying wheelchair activity in child users over an extended period of time (seven days), and Coulter et al. (2011) validated these methods even further by testing adult wheelchair users. Output from the wheel-mounted accelerometer provided detailed information about the wheel revolutions, absolute angle of velocity and duration of movement. In the Coulter et al. work, it was revealed that the vast majority of wheeled movement happens in bouts of less than 1 min. These short bursts of movement are important findings when understanding how wheelchairs are used in daily life and further supports the utility of this method.

Sonenblum et al. (2012a, 2012b) advanced the work by Cooper et al. (2008b) and Coulter et al. (2011) with the development of a multi-stepped algorithm that improves the validity of wheel-mounted accelerometer measurements. The algorithm developed in this study is able to filter out accelerations from sources other than wheel rotation (e.g. vibrations), which helps prevent overestimating duration and frequency of wheeled mobility. Results indicate that the algorithm is highly accurate in measuring when the wheelchair is actually moving, especially regarding the direction. In addition, this approach can be used to define bouts of mobility, which is a key indicator of human movement.

Wheel-mounted data logging systems or accelerometers have been used to reveal differences in wheeled mobility behaviour in a variety of conditions. This technique has uncovered different rates of acceleration associated with various activity settings (Tolerico et al. 2007), differences between boys’ and girls’ mobility characteristics during daily life activities (Cooper et al. 2008b), average length (in time) of wheeled movement (Coulter et al. 2011) and techniques for mapping wheeled mobility in terms of bouts of mobility (Sonenblum et al. 2012a, 2012b). Possibly the most critical theme associated with this line of research is the ability to evaluate wheeled mobility in applied settings (i.e. outside of a controlled setting).

In addition to wheeled mobility outcomes, it is important to continue use of established performance tests to (1) add to the growing body of literature on performance outcomes and (2) provide additional information about the effect of wheelchair design on user performance. Use of the WPT yields a straightforward propulsion efficiency (metres per cycle) score after a short test. Given the extensive literature on propulsion systems and user performance (Mukherjee & Samanta 2001a, 2001b; van der Woude et al. 2001; Winter et al. 2010, 2012), it is hypothesised that alternative propulsion systems are more efficient than push rim designs. However, this test has yet to be used in the context of evaluating wheelchair designs in less-resourced areas.

## Research methods and design

### Materials

Participants were recruited through the Centre for Medical Rehabilitation (CMR), a branch of the Ministry of Health in Lao PDR. Requirements for participation included (1) full-time use of a wheelchair, (2) functional use of the upper extremities allowing the individual to self-propel a wheelchair and (3) using a functional wheelchair manufactured at the wheelchair workshop, located within the CMR, as their primary mode for transportation.

**FIGURE 1:**
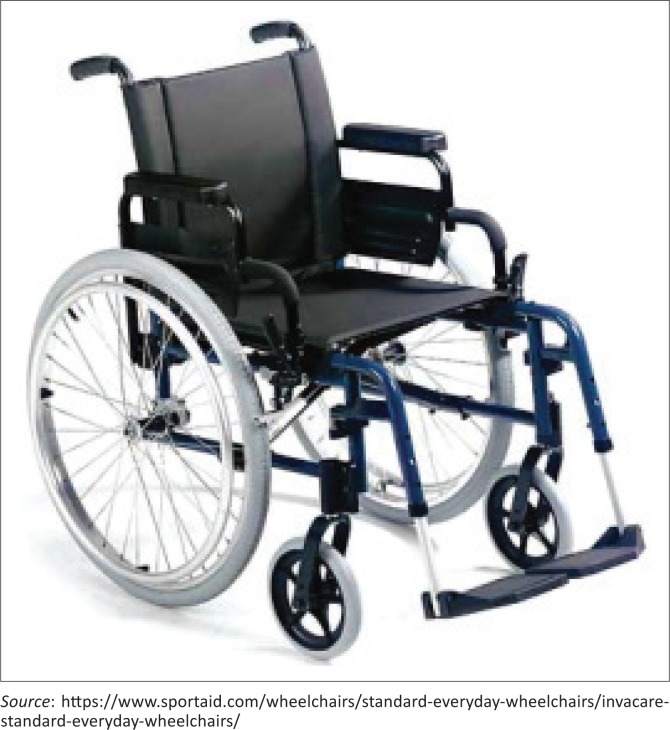
Push rim wheelchair.

**FIGURE 2:**
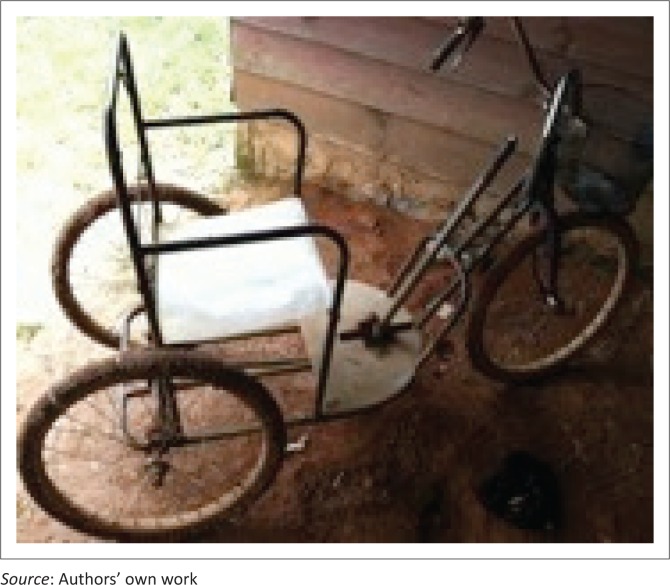
Tricycle.

CMR officials selected the wheelchair users who met the qualification criteria. Of the 18 adults who qualified, 14 individuals consented to participate and completed all stages of data collection. The four individuals who were not included in the final group were excluded because of (1) not being contactable (2) not being able to self-propel a wheelchair or (3) not having a functional wheelchair at the beginning of the project. At the beginning of the data collection, 3 participants were using a push rim wheelchair (Figure 1), whereas the remaining 11 participants were using a tricycle (Figure 2) With regard to disability type, nine were post-polio, four had undiagnosed congenital birth defects and one was paralysed from the waist down because of a motorbike accident. Five of the participants were female, and the remaining nine participants were male. The ages of the final sample ranged between 23 and 56 years. Participant characteristics are displayed in Table 1.

**TABLE 1:** Participant characteristics.

Personal wheelchair	Age	Gender	Disability
Tricycle	42	Male	Post-polio
Tricycle	54	Female	Post-polio
Tricycle	46	Male	Post-polio
Tricycle	24	Male	Congenital
Tricycle	56	Female	Post-polio
Tricycle	23	Male	Congenital
Tricycle	40	Male	Post-polio
Tricycle	37	Male	Post-polio
Tricycle	29	Male	Post-polio
Tricycle	33	Female	Post-polio
Tricycle	47	Male	Post-polio
Push rim	38	Female	Congenital
Push rim	24	Female	Congenital
Push rim	30	Male	Accident

*Source*: Authors’ own work

**FIGURE 3:**
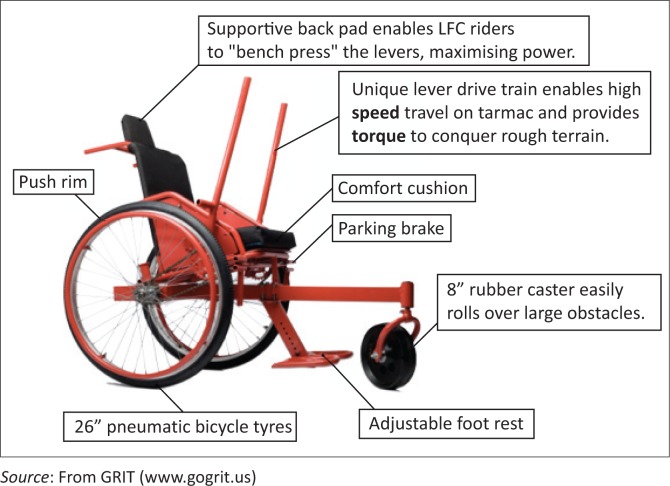
Leveraged Freedom Chair.

The alternative wheelchair used in this study was the Leveraged Freedom Chair (LFC) (Figure 3) (Global Research Innovation and Technology [GRIT] [www.gogrit.org)). The LFC is a unique design that features a dual-lever propulsion system, which enables the user to generate more mechanical power compared with other wheelchair designs (Winter et al. 2010, 2012). Leverage bars are attached to a free-wheel bicycle gear system that enables the user to generate forward propulsion strokes and maintain movement during the recovery phase, just as a typical bicycle operates. Both leverage bars are independent from one another, enabling the user to turn easily and both lever bars can be removed at any time. The LFC was developed for use in developing countries and has been tested and evaluated in east Africa (Winter et al. 2010), Guatemala (Winter et al. 2012) and India (Winter et al. 2012). GRIT donated all LFCs used in this study but no additional financial support.

### Setting

All data collected in this research took place in Xiengkhouang and Savannakhet provinces in the Lao PDR. This research was approved by the Ministry of Health in the Lao PDR and was facilitated under the supervision of World Education Laos (WEL). Wheelchair operations in the Lao PDR are facilitated through the CMR in Vientiane. All locally manufactured wheelchairs are produced at the workshop in Vientiane and then distributed to provincial rehabilitation centres (PRCs) for delivery to the patients. Data collection was facilitated under partnerships with staff members of the CMR, including the Xiengkhouang and Savannakhet PRCs.

### Design

A quasi-experimental research design was used to measure the effect of the alternative propulsion design wheelchair. Mobility was measured with a tri-axial accelerometer (Gulf Coast Data Concepts) attached to the spoke of the wheelchair (Cooper et al. 2008b; Coulter et al. 2011; Sonenblum et al. 2012a, 2012b). Mobility data were collected from all participants in two separate phases: (1) while using their personal wheelchair (PC) (i.e. locally made wheelchair) and (2) while using the provided alternative LFC.

Initial measurements were collected during an evaluation period of mobility employing the user’s PC. Mobility data were again collected after distribution of the LFC. Mobility data were collected over a 5-day period during each collection session, totalling 10 days of mobility data for each participant.

**FIGURE 4:**
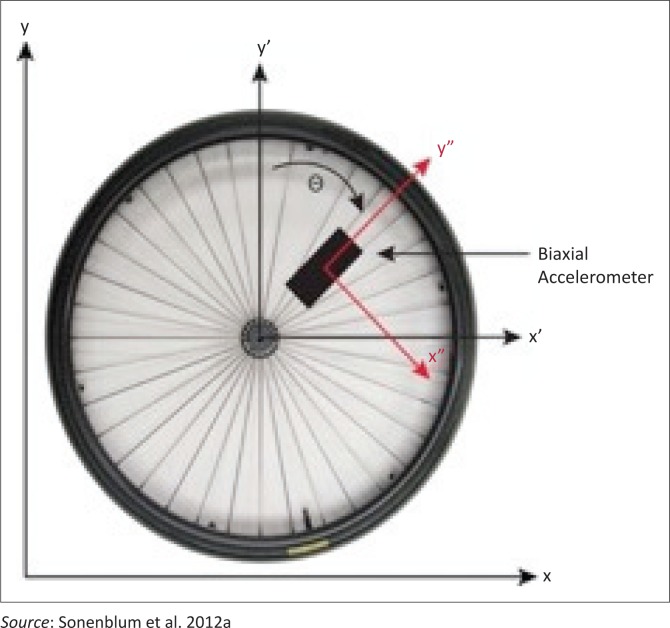
Orientation of spoke-mounted accelerometer.

The placement of the accelerometer on the spoke of the wheelchair (Figure 4) is not invasive to the user and does not change the wheelchair function. Each accelerometer was secured to the wheelchair spoke in a manner that prevented users from moving the device. Accelerometers were weatherproofed to ensure full functionality in damp or dusty conditions. Accelerometers were battery-powered and were set to collect data at a sampling rate of 12 Hz. This sampling rate was selected due the length of time data were collected (five days) and to accommodate the inevitably large data files. Each accelerometer was equipped with a USB that enabled upload of data to a computer after the data collection period was complete. All data were then uploaded to a password protection cloud. Data were de-identified with a randomly generated code assigned to each participant.

### Procedure

All participants were trained on how to use the LFC for a minimum of 1 h. During each training session, researchers ensured that the user could (1) properly insert and remove both levers, (2) complete at least 10 turns (180°) in both left and right rotations, (3) propel the chair with lever bars for a consecutive 50 m and (4) travel to and from their home on a self-selected course within 100 m of the starting point. In addition to the training period, all participants were given a 10-day acclimatisation period prior to post-intervention data collection.

The WPT (Askari et al. 2013) was administered to each participant. The WPT is used for assessing wheelchair propulsion effectiveness by recording data on velocity (ms^−1^), cadence (cycles per second) and efficiency (metres per cycle) while propelling over a pre-established distance. The WPT protocol for this study had participants propel continuously for 10 m, turn around (always to the left) and come back to the starting point (a total of 20 m). All participants were timed, and the right arm was used to count the number of cycles in accordance with WPT guidelines. The distance selected in this study (20 m) was adapted from the published recommendation of 10 m to accommodate for the 180° turn. The turning phase was used to add complexity to the test and evaluate propulsion during a turn. Authors of the assessment explain that the distance can be manipulated if desired (Askari et al. 2013). The WPT was administered at two different times during data collection: (1) during the initial visit after the accelerometer was attached to the participant’s wheelchair and (2) during the fourth (and final) visit when the accelerometer was removed from the LFC. At the time of the second WPT administration, each participant had been in possession of the LFC for at least 15 days.

All participants were visited four separate times during their involvement in the study. A detailed outline of participant visit objectives is shown in Box 1.

BOX 1 Visit-by-visit detailed objectives.

**Table:** 

Visit #1	Obtain consent; attach accelerometer to locally manufactured wheelchair; administer WPT	Conduct 1-h interview if pre-selected individuals consent to participate in interview section of the study.
**Five-day data collection period**		
Visit #2	Collect data from accelerometer; distribute all-terrain wheelchair and provide a training session on how to properly use the new wheelchair; administer WPT	
**Ten-day all-terrain wheelchair acclimatisation period**		
Visit #3	Attach accelerometer to new all-terrain wheelchair	
**Five-day data collection period**		
Visit #4	Collect data from accelerometer; administer second WPT	

*Source*: Authors’ own work

WPT, wheelchair propulsion test.

### Analyses

Raw data exported from the accelerometers was represented in X and Y displacements measured over time. These data were analysed to establish individual bouts of mobility (Sonenblum et al. 2012b) during the two separate five-day testing periods (pre- and post-intervention). Displacement data were analysed via a five-step algorithm (Sonenblum et al. 2012b), which indicated whether or not the wheelchair was moving (forward or backward). This analysis only requires the use of the X and Y coordinates, representing tangential and radial orientation, respectively. The ‘moving threshold’ for this study was defined at 0.12 ms^−1^, consistent with previous research (Sonenblum et al. 2012b). Displacement above the threshold was defined as a bout of mobility.

Total bouts were added over the five-day collection period for each participant, and the day with the most bouts (i.e. peak bouts) was used for comparative analysis. Peak bouts over each five-day testing period were recorded to help normalise the representative data and avoid heavily skewed and/or inconsistent bouts detected over the collection period. The WPT score, collected before and after LFC use, was used to define propulsion efficiency.

A repeated measures analysis of variance (ANOVA) was used to test for statistical differences between and within conditions. Time specified pre- and post-intervention testing periods. The same statistical test was used to detect differences for all three dependent measures: total bouts of mobility over 5-day testing periods, peak bouts of mobility during 5-day testing period and propulsion efficiency (i.e. WPT). Levene’s test was used to examine homogeneity of samples. Significance was determined by *p* values < 0.05 and all statistical analyses were conducted via SPSS. Descriptive statistics are also reported for comparative analyses.

## Results

The following results, defining the wheeled mobility and propulsion characteristics explored in this study, are presented. Table 2 provides descriptive statistics for each individual participant across all dependent variables measured with the individual’s PC and LFC. Data are separated by PC type and organised, in descending order, by total bouts accumulated during PC use.

**TABLE 2:** Individual participant descriptive statistics for performance measures.

WHC type	Total bouts (PC)	Total bouts (LFC)	Avg. bout length (s) (PC)	Avg. bout length (s) (LFC)	Total active time (h) (PC)	Total active time (h) (LFC)	Peak bouts (PC)	Peak bouts (LFC)	WPT (metre per cycle) (PC)	WPT (metre per cycle) (LFC)
Tricycle	534	537	42.12	71.61	6.25	10.68	172	241	1.33	1.11
Tricycle	481	234	87.88	41.33	11.74	2.68	189	97	1.43	0.67
Tricycle	295	7	58.39	68.89	4.78	0.13	179	6	0.87	0.59
Tricycle	277	42	42.51	80.49	3.17	0.94	99	15	1.43	0.87
Tricycle	211	76	60.60	72.43	3.55	1.53	106	31	1.54	0.54
Tricycle	94	78	278.84	168.02	7.28	3.64	51	42	1.25	0.87
Tricycle	93	94	27.70	36.10	0.72	0.94	36	46	1.00	0.50
Tricycle	88	115	221.84	64.36	5.42	2.06	44	53	1.54	0.95
Tricycle	68	38	77.82	72.19	1.47	0.76	33	14	1.67	0.35
Tricycle	68	89	22.91	23.14	0.43	0.57	23	33	1.33	1.11
Tricycle	31	18	62.03	51.59	0.53	0.26	19	17	1.54	0.95
Push rim	648	451	29.72	33.84	5.35	4.24	259	199	0.67	1.11
Push rim	81	372	65.15	60.17	1.47	6.22	35	167	0.39	1.05
Push rim	48	31	48.95	65.04	0.65	0.56	27	18	0.27	0.69

*Source*: Authors’ own work

LFC, Leveraged Freedom Chair; PC, personal wheelchair; WPT, wheelchair propulsion test.

### Total bouts of mobility

Results from the summation of bouts of mobility accumulated over the five-day testing period reveal no differences between locally manufactured wheelchairs and LFCs (*p* > 0.05). Table 3 displays descriptive statistics for all wheeled mobility dependent variables across all participants. Results from Levene’s test indicated that the assumption of homogeneity was not violated, suggesting there was equality of variance between participants (*p* > 0.05).

**TABLE 3:** Wheeled mobility results reflecting means and standard deviations (*N* = 14).

Group	Personal chair	LFC
Total bouts	215.5 (**203.59**)	155.86 (**173.47**)
Bout length (s)	80.47 (**75.17**)	64.94 (**34.35**)
Active time (h)	3.77 (**3.28**)	2.52 (**2.94**)
Peak bout length	90.86 (**78.28**)	70.64 (**76.12**)

*Source*: Authors’ own work

Data in bold are the standard deviations.

LFC, Leveraged Freedom Chair.

Two additional secondary analyses, average bout length and total active time, were conducted to further investigate the bout characteristics over the testing periods. Both analyses were conducted using the same statistical analysis: repeated measures ANOVA. In the first analysis, average bout length (s) was calculated and there were no differences between testing periods (*p* > 0.05). This analysis was conducted to reveal the average length of time each participant was sustaining wheeled movement. Results from Levene’s test indicated that the assumption of homogeneity was not violated, indicating there was equality of variance between groups (*p* > 0.05). Just as was seen in the total bouts accumulated analysis, there was a large amount of variation (i.e. high standard deviations) within groups.

In the second analysis, time spent in motion [i.e. active time (h)] was calculated, and there were no differences between testing periods (*p* > 0.05). This analysis was conducted to display changes in gross activity level over both testing periods. Levene’s test indicated that the assumption of homogeneity was not violated, again, indicating there was equality of variance between groups (*p* > 0.05).

Analysis of peak bouts accumulated over a single 24-h period revealed no differences when using the LFC (*p* > 0.05) across testing periods for all participants. Just as was seen with previous analyses, there was a high level of variation within groups, but Leven’s test again indicates that there was homogeneity (*p* > 0.05).

### Propulsion efficiency

Results from the WPT (Table 4) revealed no differences in propulsion efficiency between locally manufactured wheelchairs and LFCs (*p* > 0.05). Results from Levene’s test indicated that the assumption of homogeneity was not violated (*p* > 0.05). A secondary analysis was conducted on propulsion efficiency to uncover how an individual’s PC (i.e. tricycle or push rim) influenced his or her transition to the LFC. This analysis was conducted as a result of participatory observation that tricycle users struggled with the transition to the LFC, whereas push rim users experienced a smooth transition. This analysis was conducted using a 2 (group) × 2 (time) repeated measures ANOVA with a Bonferroni adjustment for multiple comparisons and identification of group interactions. Although separation into groups based on PC yielded unbalanced sample sizes for tricycle (*N* = 11) and push rim (*N* = 3) users, the repeated measures approach helps control for individual differences and unequal sample sizes.

**TABLE 4:** Average propulsion efficiency (metres per cycle) during WPT (SD).

Group	Personal chair	LFC
Overall (*N* = 14)	1.16 (**0.45**)	0.81 (**0.25**)
Tricycle (*N* = 11)	1.36 (**0.24**)^a^	0.77 (**0.26**)^a^
Push rim (*N* = 3)	0.44 (**0.21**)^a^^,^^b^	0.95 (**0.23**)^a^

*Source*: Authors’ own work

Data in bold are the standard deviations.

LFC, Leveraged Freedom Chair.

a , Significant differences within groups.

b , Significant differences between groups.

**FIGURE 5:**
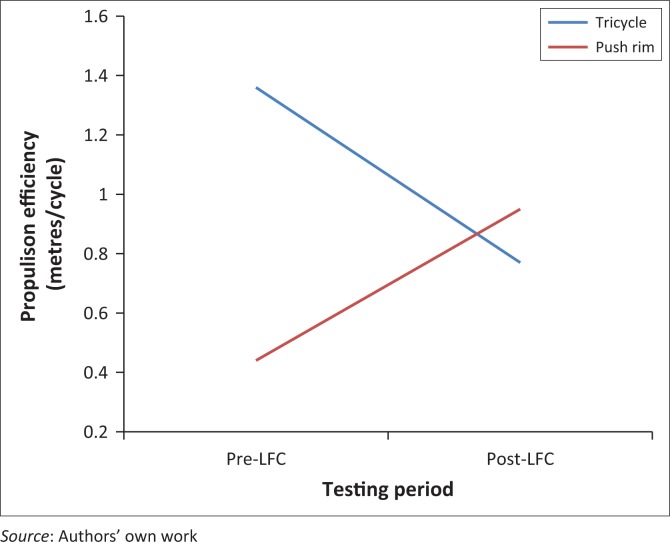
Changes in propulsion efficiency (metres per cycle) before and after LFC use.

Results revealed significant difference both between and within groups. For participants who started using a tricycle, their propulsion efficiency significantly decreased (*p* < 0.001) when using the LFC, which was a large effect (η^2^_p_ = 0.76). Conversely, participants who started using a push rim wheelchair experienced significantly higher propulsion efficiency (*p* < 0.05) when using the LFC, which was a moderate effect (η^2^_p_ = 0.39). A significant interaction was detected between group and testing period (*p* < 0.001), which was a large effect (η^2^_p_ = 0.70) (Figure 5). When comparing outcomes between groups, results indicate that tricycle users had significantly higher efficiency values (*p* < 0.001) compared with push rim users prior to LFC use, which was a large effect (η^2^_p_ = 0.75). There was not a significant difference between groups when using the LFC (*p* > 0.05), but push rim users exhibited higher efficiency values.

### Potential benefits and hazards

None of the participants was at abnormal risk as a result of participation in this study. The LFC provides no additional risk to the user, and all participants kept their LFCs at no cost. All data were immediately de-identified, and none of the information collected during this research was made available to anyone outside of the research team until it was de-identified.

### Recruitment and informed consent

All participation in the study was voluntary and subjects were recruited from a list of qualified individuals, which was created by the CMR in Vientiane, Lao PDR. Informed verbal consent was received and recorded from all participants. Written consent (i.e. signatures) was not obtained because of the low literacy rate in areas of Laos where data were collected. All participants were told that they could end their involvement at any time during the study.

## Trustworthiness

### Reliability

The results of this study are considered reliable because of the rigorous process in selecting a subject population. All participants were selected on the basis of their wheelchair dependency and daily activity, as defined by an understanding of their daily routines, travel distances within their community and previous experiences as a wheelchair user. In addition to the homogeneous population, the methodology in the study is clearly defined and highly repeatable, given the amount of detail provided. Repeatability of this research is not specific to location (i.e. Laos), but would require an active wheelchair user population, who are inexperienced with dual-level propulsion systems, and given the same amount of time to acclimatise to the new product.

### Validity

The results of this study are considered valid because of the previous research that supports the use and validity of the two evaluation methods: WPT and bouts of mobility. Data collected under both evaluations were complete and analysed in line with previous work. Inconsistencies with previous research can be a result of wheelchair designs tested, physical setting, subject population and acclimatisation time period, all of which are unique to this particular study.

## Discussion

### Outline of the results

Results indicate there were no differences between PC (i.e. tricycle and push rim) and LFC use. The high level of variability between subjects can explain the lack of differences detected across all dependent variables, both with and without the LFC. Dramatically high standard deviations seen across all outcomes make the data difficult to interpret. Although no differences were found in propulsion efficiency across all participants, when separated into groups by PC design, differences were detected. Statistical evidence indicated that tricycle users did not benefit, in terms of efficiency, from using an LFC, while push rim chair users experienced an improvement in efficiency when using the LFC. These findings support existing literature that lever-drive propulsion systems are more efficient compared with push rim systems.

The significant interaction effect detected between tricycle and push rim users across propulsion efficiency testing period provides further explanation about the differences uncovered. Tricycle users’ ability to be significantly more efficient than push rim users prior to LFC is supported by previous research (Mukherjee & Samanta 2001b; Van der Woude et al. 2001). In addition, results indicate an easier and beneficial transition to the LFC for push rim users, which could be explained by the bimanual propulsion methods employed by both designs.

Interesting mobility characteristics were revealed when examining influence of wheelchair design at the individual level. For example, one subject, a tricycle user, experienced an increase in total bouts, but a dramatic decrease in average bout length. This suggests that although this participant exhibited a higher frequency of movement (i.e. bouts) with the LFC, their ability to sustain continuous movement (i.e. bout length) decreased, which also led to the decrease of active hours. Bout characteristics for another tricycle user provided another telling example how these data can be interpreted. This subject had a slight increase in total bouts (difference of +3), but displayed a large increase in sustained movement (i.e. bout length) and active time with the LFC. These outcomes suggest that the LFC enabled an already active individual to sustain longer bouts of activity, which ultimately promoted a longer duration of activity over the testing period (i.e. active time).

As for the three push rim users, two exhibited a decrease in total bouts with the LFC, yet increased their average bout length. This suggests that the LFC was easier to use for these two participants, which increased their time of sustained movement (i.e. bout length), but did not encourage an increase in active time and, therefore, decreased overall frequency (i.e. total bouts). In addition, one push rim user experienced the highest increases in total bouts, total active time, peak bouts and propulsion efficiency across all participants, regardless of personal chair design.

Although no differences were found in average bout length with and without the LFC, results can still be compared to the Coulter et al. (2011) study. Interestingly, average bout length was more than 60 s across all participants with and without the LFC. This is contrary to the Coulter et al. findings, which described the majority of wheeled movement lasting less than 60 s at a time. The nature of bout length among wheelchair users in developed and developing countries should be investigated further.

Use of peak bouts helped normalise participants’ mobility behaviour by signifying the distribution of bouts over the five-day testing period, which helped identify heavily skewed data. For example, one subject accumulated a total of 18 bouts while using the LFC and 17 of those bouts occurred during a single 24-h period (i.e. peak bouts). When considering this participant’s average bout length with the LFC was 51.59 s, the peak bout results suggest that this individual used the LFC for only a short period of time and then possibly abandoned the device. Identifying peak bouts helps establish how consistent, or inconsistent, wheeled mobility is over the given testing period and this variable should continue to be used in future research.

### Practical implications

Results from this study highlight the benefits of using wheel-mounted accelerometers to measure wheeled mobility over an extended period of time. This is the first study to use this method in context of understanding mobility in a developing country. This study also serves as an example for using a performance test (e.g. WPT) along with an extended mobility assessment to build a comprehensive understanding of wheelchair use. Benefits associated with propulsion systems were confirmed during this research, and we also learned about the barriers associated with transitioning to a different wheelchair design (e.g. tricycle users to the LFC). Overall, this study reaffirms the importance of wheelchair prescription and patient assessment in order to ensure users are given products that best fit their everyday needs.

## Limitations of study

Although the sample size for this study was relatively low, moderate effect sizes were seen across propulsion efficiency outcomes. Previous research indicates that recruitment is an inherent limitation when collecting data in less-resourced areas (Mukherjee & Samanta 2001a, 2001b, 2005; Winter et al. 2010, 2012). Recruitment in these settings is determined primarily by access to qualified participants and travel time, which is driven by financial resources. Although these barriers exist, the methods and outcomes in this study suggest moderate effect sizes can be produced during a test, such as the WPT. It can also be argued that the sampled population in this study is not representative of all wheelchairs users in the Lao PDR. This is because all participants were recruited from the rehabilitation centre’s database, which does not have patient records on every wheelchair user in the country.

### Recommendations

Future research can investigate these transition dilemmas in a variety of ways. One approach is to only study changes in wheeled mobility for push rim users transitioning to an LFC. Given the similarities in baseline propulsion mechanics (i.e. bimanual asymmetric movements), this research design would focus directly on the effect of the LFC. Another approach, for measuring tricycle users’ ability to transition to the LFC, would be to increase the duration and frequency of LFC practice sessions during the acclimatisation period. These research designs would also benefit from prolonged acclimatisation periods, which could help decrease variability between participants and lead to more normalised data samples.

Difficult transitions seen by some participants, namely tricycle users, also led to a lack of interest in using the LFC. All participants in this study depend on wheelchairs as their primary source of mobility, and their livelihood often depends on their ability to move efficiently. Those who experienced a difficult transition to the LFC most likely abandoned the new wheelchair immediately and reverted to their personal chair because of the familiarity with the older product. Considering human subjects’ research will not permit taking the personal chair away from the participant to ensure they use a new product, the focus must be directed towards an increased frequency of training sessions to facilitate a smoother transition.

## Conclusion

Outcomes generated from the wheeled mobility and propulsion efficiency tests offer valued information on how individuals interact with different wheelchair designs. Dependent measures revealed unique individual characteristics about mobility and results support the benefits of using the combination of WPT outcomes and wheeled mobility (i.e. bouts of mobility) as effectiveness measures. More specifically, this research shows that wheel-mounted accelerometers and bouts of mobility outcomes can successfully be used in rural settings over an extended period of time. This is the first study to uncover great detail on wheeled mobility characteristics during an individual’s daily activities in a less-resourced setting.

When considering this was the first study to use this method to compare different wheelchair designs in these particular environments, this research should be considered a pilot design. The robust wheeled mobility data revealed from this study sets the stage for meaningful future research. It is clear that some individuals benefitted from the LFC, while others did not, and the root causes of this phenomenon need to be investigated further. For example, the type of baseline wheelchair (i.e. personal chair) seems to affect how an individual transitions to the LFC. This can be explained by the similarities and differences in design between the three different propulsion systems. Both the push rim chair and LFC require bimanual, asymmetric upper-limb movements to move and control the wheelchair. Conversely, the tricycle requires one arm to generate power and one arm to control the wheelchair. Because the propulsion motions are different between the tricycle and LFC, tricycle users experienced a difficult transition when using the LFC. The similarities in bimanual asymmetric propulsion seen in push rim and LFC use helped push rim users adapt quickly to the LFC and immediately experience improvements in efficiency.

This paradigm can be explained by learning differences associated with propulsion systems through the examination of skill complexity and focus of attention. Skill complexity, overall, is increased with use of the LFC, as it is a unique method of propulsion; however, push rim users are already familiar with a bimanual propulsion system, resulting in a smoother transition. Therefore, push rim users experienced a significant increase in propulsion efficiency when using the LFC. The bimanual aspect of LFC use served as a barrier for tricycle users and resulted in significantly lower propulsion efficiency.

Transition to the LFC, for tricycle and push rim users, also supports the constrained-action hypothesis (McNevin, Shea & Wulf 2003; Wulf, McNevin & Shea 2001), which establishes the importance of focus on movement execution. For example, it was observed during the third and fourth participant visits that tricycle users were focused primarily on the leading castor or levers on the LFC, rather than looking forward and completing strong propulsion strokes. This observation could suggest that tricycle users were typically focusing on movements, rather than movement effect, which interfered with their motor control processes. Conversely, the improved transition seen by push rim users can be explained by their focus on the movement effect and overall comfort with LFC use. These principles highlight the importance of effective and sustained education for patients transitioning to different wheelchair propulsion designs, which should be completed through frequent, consistent practice in variable conditions.

These conclusions focus attention on the importance of adequate and effective wheelchair prescription and utilisation of various wheelchair designs. This study yields information that truly informs practitioners about how wheelchairs are used and how patients interact with various products. Future research should continue the expansion of application for wheel-mounted accelerometers and the robust information they can produce, such as wheeled mobility. These approaches will continue to tell us how patients interact with wheelchairs in their personal environments and aid practitioners in providing improved and comprehensive services.
